# Electroencephalographic Response in Juvenile Tambaqui, *Colossoma macropomum*, Exposed to Short-Term Anaesthetic Baths with Geraniol and Citronellol

**DOI:** 10.3390/biology12010090

**Published:** 2023-01-06

**Authors:** Ednara Ronise Lima De Araújo, Marcelo Ferreira Torres, Brenda Maria Pereira Alho Da Costa, Moisés Hamoy, Luís André Sampaio, Luis André Luz Barbas

**Affiliations:** 1 Laboratório de Piscicultura Estuarina e Marinha, Programa de Pós-Graduação em Aquicultura, Instituto de Oceanografia, Universidade Federal do Rio Grande, CP 474, Rio Grande 96201-900, Brazil; 2 Laboratório de Aquacultura de Espécies Tropicais, Instituto Federal de Educação Ciência e Tecnologia do Pará, Castanhal 68740-970, Brazil; 3 Laboratório de Farmacologia e Toxicologia de Produtos Naturais, Instituto de Ciências Biológicas, Universidade Federal do Pará, Belém 66075-110, Brazil

**Keywords:** electroencephalography, monoterpenes, anaesthetic, sedation, fish welfare

## Abstract

**Simple Summary:**

Anaesthetics have been increasingly used for live fish manipulation to facilitate management, reduce stress and for welfare purposes. Plant derived natural compounds have been recommended as alternatives to synthetic drugs. Herein, juveniles of tambaqui fish, *Colossoma macropomum*, were exposed to geraniol and citronellol, which have been proposed as promising natural products for fish anaesthesia. The electroencephalographic response was characterised upon short-term exposure of fish to both compounds in anaesthetic concentrations, and throughout recovery in clean water. While geraniol-exposed fish showed an adequate anaesthetic effect, with depression of the central nervous system and a gradual recovery of the brain electrical activity, fish exposed to citronellol had an altered electroencephalographic response during induction, somewhat incompatible with an appropriate anaesthetic effect and smooth recovery.

**Abstract:**

The aim of this study was to evaluate the level of neuronal depression in juvenile tambaqui, *Colossoma macropomum*, exposed to geraniol (GRL) and citronellol (CTL) in immersion baths. A total of 36 juveniles weighing 35.2 ± 9.4 g were used, organised into six experimental groups: I—control (clean water); II—ethanol (water containing the highest volume of ethanol used in the anaesthetic pre-dilution); III—GRL induction (70 µL·L^−1^); IV—CTL induction (90 µL·L^−1^); V—GRL recovery; VI—CTL recovery. Electroencephalographic (EEG) recordings were performed for 300 s in each group. EEG tracings of the control and ethanol groups showed regular and similar activity. Upon exposure to the anaesthetics, irregularities were observed in the tracings showing neuronal excitability and increased amplitudes, mainly in the case of CTL. Overall, GRL-exposed fish showed depression of the central nervous system with low and regular tracings throughout induction, presenting a gradual recovery and stable tracings, which were consistent with an adequate general anaesthetic effect. On the other hand, fish exposed to CTL showed altered EEG activity during induction, that could be considered incompatible with an appropriate anaesthetic effect and smooth recovery, presenting high and irregular EEG tracing amplitudes.

## 1. Introduction

Growing research efforts have been directed toward the evaluation of anaesthetics for use in aquatic animals; however, only a few studies have used an in-depth approach, e.g., to evaluate the extent of the central nervous system (CNS) depression attained, as well as other possible neuronal-related effects on the fish brain exposed to anaesthesia. Recent studies have used a combined brain–behaviour perspective to assess fish anaesthesia throughout and after exposure, whereby not only is visual or behavioural assessment considered for the characterisation of the anaesthetic suitability, but so is the modulation of the brain activity, which may reflect the efficacy of the product as a general anaesthetic or reveal important and limiting side-effects that otherwise would not be detected exclusively by visual evaluation [1,2].

Synthetic drugs such as quinaldine sulphate, benzocaine, and tricaine methanesulphonate (MS-222) have long been the most used anaesthetics for fish, despite reports of some undesirable side-effects such as irritability, loss of mucus, corneal damage, and contradictorily, physiological stress [3,4,5,6,7], with disruption of homeostasis, consequently affecting fish performance and health.

Moreover, several natural plant-derived products have been proposed for use in fish, although some of them have elicited undesirable side-effects [8]. Behavioural and neuronal changes were observed in juvenile tambaqui, *Colossoma macropomum*, upon short-term exposure to eugenol in baths, in which an intense neuronal excitability emerged, resembling a convulsive event and without depression of the CNS as shown by the EEG tracing patterns recorded from the midbrain [1]. It has been reported that eugenol, which is a traditional natural product for fish anaesthesia worldwide, might not be a suitable product for short-term anaesthetic induction of fish, as it could imply important concerns in terms of fish welfare. Therefore, alternative products should be continuously prospected for use in fish, not only relying on their capacity to promote full body immobilisation, but also considering their suitability from a neuronal perspective.

In any case, the use of natural products for sedation (light anaesthesia) or deep anaesthesia has increased in an attempt to reduce the stress levels in fish farming, while ensuring increased survival, yields, and welfare, and complying with ethics-related issues on the use and handling of live fish for farming or research purposes [8,9]. Among the natural compounds recently explored for use as fish anaesthetics are the phytochemicals geraniol and citronellal, which have presented promising sedative and anaesthetic properties. These are the main monoterpene constituents within the citronella grass, *Cymbopogon nardus* essential oil [10], and they have already been investigated for their effects on fish behaviour, skeletal muscle contraction power, and cardiorespiratory response [11,12]. Yet, additional information is required on the ability of these compounds to promote adequate CNS depression, devoid of any neuronal hyperexcitability or seizure-like patterns, thus lending more credence to their use as safe general anaesthetics for fish. 

Despite the variety of studies on novel anaesthetics for fish, mainly natural products such as essential oils and isolates [8], the characterisation of the CNS response and possible deleterious effects on brain function are still poorly elucidated for fish [13]. To overcome this limitation, the monitoring of anaesthesia should include appropriate and selected parameters. In this sense, electrophysiological analyses can be used to validate protocols, as well as reinforce the objectivity of the general anaesthesia evaluation process [1,11]. 

Several studies on the anaesthesia of juvenile tambaqui have already been reported. This freshwater fish species shows resistance to handling and high sensitivity to the testing of anaesthetic candidates. It has been considered a promising experimental model in electrophysiological studies according to recent studies [1,2,11,12,14,15,16,17].

Thus, the objective of this study was to evaluate, through electroencephalographic recordings, the level of neuronal depression in the fish brain exposed to short-term immersion baths with geraniol and citronellol, using juveniles of tambaqui, *C. macropomum*, as live models.

## 2. Material and Methods

### 2.1. Experimental Fish

The procedures described in this study were approved by the Animal Ethics Committee of the Federal Institute of Pará/IFPA—Castanhal—Protocol #6686081118 (ID 000021).

Juveniles of tambaqui (~100 fish), weighing 35.2 ± 9.4 g, were purchased from a commercial fish farm. Upon arrival, the animals were acclimated for 7 days in 300 L tanks at the Laboratory of Pharmacology and Toxicology of Natural Products, Universidade Federal do Pará (UFPA). No mortality occurred throughout or after transportation. The fish were maintained under constant aeration and photoperiod set at 12 h L/12 h D. Feeding was provided twice a day with commercial feed (32% CP) until satiety. Daily cleaning was performed with partial water change (approximately 20% of the tank volume) to remove uneaten feed and faeces. The water quality variables were monitored daily and maintained as follows: temperature, 26.8 °C; pH 7.5; DO >5.0 mg·L^−1^; ammonia 0.1 mg·L^−1^.

### 2.2. Experimental Design

A total of 36 fish were randomly selected among those initially purchased and organised as follows: (a) control, in compound-free water; (b) ethanol control, in water containing the highest equivalent volume of ethanol used in the anaesthetic dilution; (c) geraniol (GRL) induction, immersion bath in water containing 70 µL·L^−1^ GRL; (d) citronellol (CTL) induction, immersion bath in water containing 90 µL·L^−1^ CTL; (e) GRL recovery; (f) CTL recovery. Nine fish were used in each group (*n* = 9), except for the recordings from recovering fish that were carried out on the same groups of fish exposed to GRL and CTL immediately after their transfer to anaesthetic-free aquarium. Each animal was considered a replica and used only once. Analyses of the electroencephalographic (EEG) recordings were carried out for 300 s during induction and recovery. 

Initially, for the confirmation of the anaesthetic-like effect of GRL and CTL, fish were submitted to immersion baths until reaching full body immobilisation, i.e., decreased opercular movements with complete loss of the postural reflex and absence of response to a tail pinch (all within 5 min). The exposure concentrations used herein and procedures followed recommendations of a previously published study [11]. 

For the assessment of the tambaqui CNS activity, the power spectral density was evaluated over a period of 5 min; the last 30 s of the induction records were also looked into, and arbitrarily chosen, for a more detailed analysis [geraniol (GRL—30 s) and citronellol (CTL—30 s)] of the onset of a presumably deeper anaesthetic plane.

The compounds (in the form of oils) used in this study were purchased from a commercial establishment (AROMACH ingredients™—Campinas, São Paulo, Brazil). Initially, a stock solution was prepared by pre-diluting the oils in ethanol (96%) in a 1:9 ratio. Later, aliquots were used for EEG evaluation. 

### 2.3. Implantation of the Electrodes and Acquisition of EEG Recordings

For the EEG recording procedure and analyses, methods previously validated and reported by our research group were used [1,18]. Briefly, for the implantation of the electrodes, after the acclimation period, fish were anaesthetised with propofol 2% (Claris Produtos Farmacêuticos do Brasil Ltd.a, Barueri, São Paulo, Brazil) at 4.0 μL·L^−1^ [15] via continuous bucco-branchial flow, by gravity, and a tail pinch was applied to check if the fish were unresponsive. Thereafter, a dental drill was used to access the brain. The reference and registration electrodes were made of stainless-steel rods and their tips (Ø 1 mm) were positioned on the mesencephalic region (midbrain), 2 mm away from each other and affixed with self-curing epoxy resin. The right electrode was responsible for the acquisition of the record, while the left electrode served as a reference to the amplifier.

After the surgery for the affixation of the EEG electrodes, fish were transferred to maintenance tanks for recovery, where they remained for 48 h prior to the measurements. Upon recordings, fish were taken according to their respective treatment and gently restrained to a slotted foam pad, inside an aquarium previously added by the respective test substance, and then the electrodes were connected from the fish midbrain (mesencephalic region) to a high-impedance amplifier for the measurement of the electric field potential in the midbrain area. After 10 s of the transfer to the induction aquarium, recordings commenced and lasted 5 min (300 s) for each animal. Subsequently, fish were immediately transferred to anaesthetic-free water in a recovery aquarium, and EEG tracings were recorded for five more minutes. After recordings, all the implanted fish were killed with a blow to the head followed by destruction of the brain. For more details on the surgery and EEG recording procedures and setup, refer to our previously reported method [1]. 

### 2.4. Signal Analysis and Statistics

A high-impedance amplifier (Grass Technologies, P511), adjusted with low- and high-pass filtering (0.3 and 300 Hz), with 5000× amplification and coupled to an oscilloscope (Protek, 6510) was used. The recordings were carried out inside a Faraday cage (TMC™); data were continuously monitored at 1 kHz range (National Instruments, Austin, TX) and analysed using LabVIEW Express software. 

For the analysis of the acquired signals, a tool was built using the Python programming language version 2.7. Numpy and Scipy libraries were used for math processing and Matplolib library for the graphics. The graphical interface was developed using the PyQt4 library [19]. Frequency spectrograms were calculated using a Hamming window of 256 points (256/1000 s), and each frame was generated with an overlap of 128 points per window. For each frame, the power spectral distribution (PSD) was calculated using the Welch mean periodogram methodology. Frequency histograms were made using the PSD (with 1 Hz boxes).

Kolmogorov–Smirnov and Levene tests were used to check data normality and variance homogeneity, respectively. Comparisons of mean power values were performed using one-way analysis of variance (ANOVA), followed by Tukey’s post hoc test. GraphPad Prism^®^ 8 software was used for the analyses. A *p*-value < 0.05 was considered statistically significant in all cases.

## 3. Results

**Figure 1:**
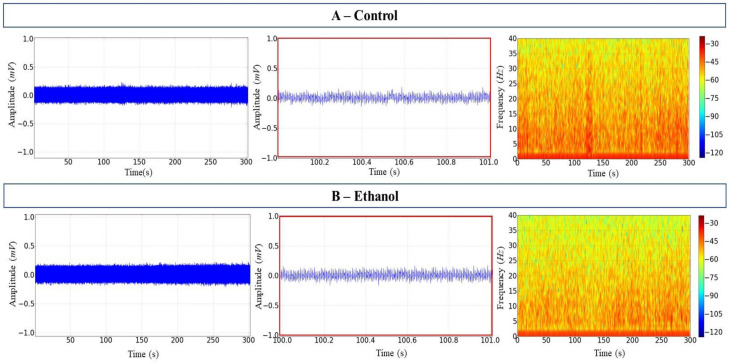
Tambaqui *C. macropomum* midbrain electroencephalographic (EEG) records. (**A**) Control records over 300 s (left); 1 s snapshot amplification (centre); the spectrogram of frequency showing the distribution of power (right). (**B**) Ethanol records over 300 s (left); 1 s snapshot amplification (centre); the spectrogram of frequency showing the distribution of power (right). Amplification of 5000×. Records from one fish only in each group.

There were no mortalities during or after the handling of the animals. The EEG recordings for the control and ethanol groups showed regular and similar activity, as can be seen in the tracings, with low mean amplitudes of approximately 0.11 ± 0.001 mV (Figure 1A,B, left and centre). The spectrogram of frequency shows the distribution of power (intensity of the signal in colour scale) in frequencies up to 40 Hz. Higher intensity was concentrated in frequencies below 5 Hz in both the control and the ethanol groups (Figure 1A,B, right).

**Figure 2:**
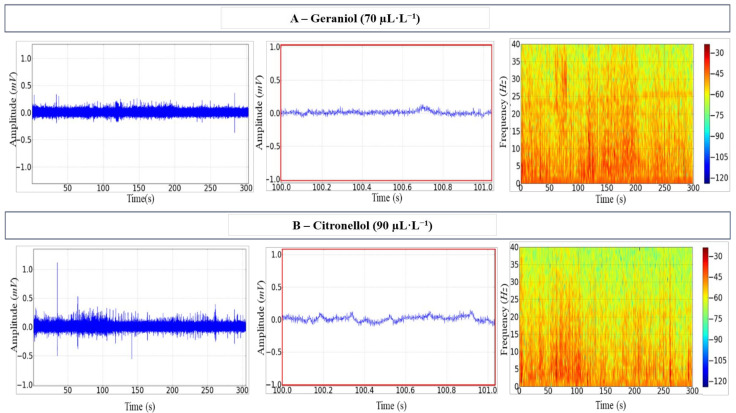
Electroencephalographic (EEG) recordings of the Tambaqui *C. macropomum* midbrain during induction. (**A**) The 70 µL·L^−1^ geraniol records over 300 s (left); 1 s snapshot amplification (centre); the spectrogram of frequency showing the distribution of power (right). (**B**) The 90 µL·L^−1^ citronellol records over 300 s (left); 1 s snapshot amplification (centre); the spectrogram of frequency showing the distribution of power (right). Amplification of 5000×. Records from one fish only in each group.

Upon exposure to either anaesthetic, irregularities were observed in the tracings, which reflected some degree of neuronal excitability with increased amplitudes. Tracing amplitude in animals submitted to GRL reached 0.23 ± 0.001 mV, and the spectrogram of frequency showed a more intense distribution of power up to around 25 Hz over time (Figure 2A).

Excitability also occurred in fish exposed to CTL during induction, with amplitude reaching 0.37 ± 0.002 mV. These fish showed a more evident and greater irregularity in the tracings (Figure 2B, centre). The respective frequency spectrogram showed the signal intensity variation during exposure to CTL (Figure 2B, right).

**Figure 3:**
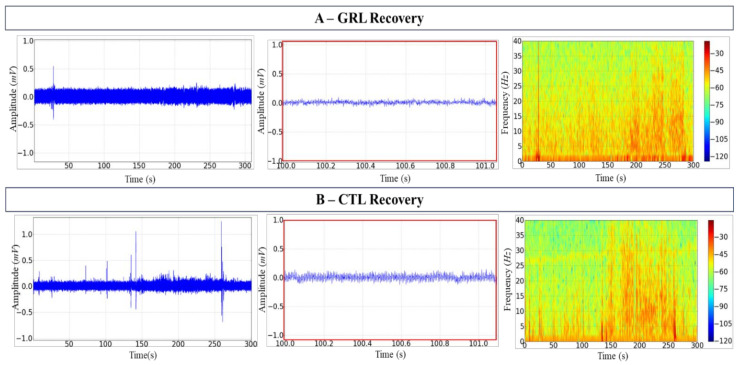
Electroencephalographic (EEG) recordings of the tambaqui *C. macropomum* midbrain during recovery. (**A**) Geraniol (GRL) records over 300 s (left); 1 s snapshot amplification (centre); the spectrogram of frequency showing the distribution of power (right). (**B**) Citronellol (CTL) records over 300 s (left); 1 s snapshot amplification (centre); the spectrogram of frequency showing the distribution of power (right). Amplification of 5000×. Records from one fish only in each group.

The recovery of fish exposed to GRL was gradual as shown by the tracings with amplitude reaching 0.25 ± 0.002 mV. Amplification of the record and the frequency spectrogram demonstrated a progressive increase in power at frequencies up to 40 Hz (Figure 3A).

On the other hand, during the 5 min recovery of fish exposed to CTL, the mean amplitude was 0.37 ± 0.001 mV; similarly to the induction, irregularities were observed in the tracings, evidenced by the amplification (Figure 3B, centre). The spectrogram of frequency demonstrated the power variation in frequencies up to 40 Hz, with a slightly higher power between 25–30 Hz (Figure 3B, right).

**Figure 4:**
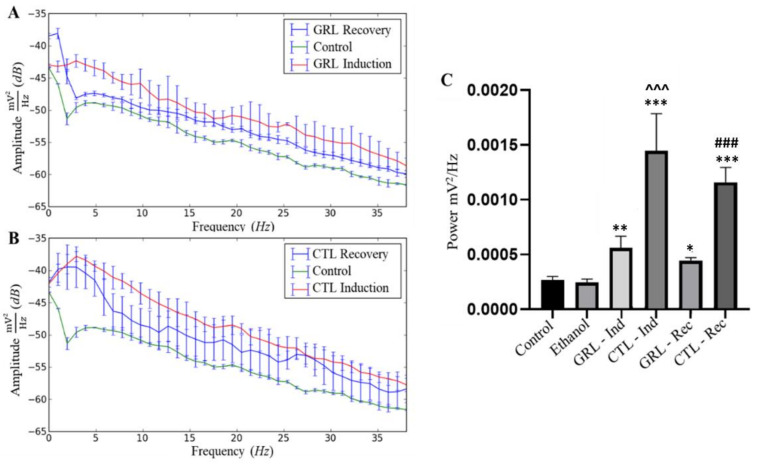
Power spectral density (PSD) of frequencies up to 40 Hz in juvenile tambaqui *C. macropomum* with comparisons among the control, during induction with geraniol (GRL—70 µL·L^−1^) and respective recovery (**A**); and during induction with citronellol (CTL—90 µL·L^−1^) and recovery (**B**). Differences in mean power among the control, ethanol, geraniol induction (GRL—Ind), citronellol induction (CTL—Ind), geraniol recovery (GRL—Rec), and citronellol recovery (CTL—Rec) groups are presented (**C**). * indicates statistical difference from the control group; ^ indicates statistical difference from the GRL—Ind group; # indicates statistical differences from the GRL—Rec group (after ANOVA followed by Tukey's test). The single * represents *p* < 0.05; Symbols in duplicate ** correspond to *p* < 0.001; Symbols in triplicate ***, ###, ^^^ correspond to *p* < 0.0001, (n = 9).

Both GRL and CTL led to higher mean power compared to the control, indicating some excitability upon contact with either substance. As for the total recording time, during recovery for both the GRL and the CTL groups, excitability was also present, albeit showing mean amplitudes below the induced group. Yet, only the PSD of the GRL-exposed fish resembled that of the control in recovery (Figure 4A,B).

The linear power distribution presented by the power spectral density (PDS) (Figure 4C) in the control and ethanol groups presented respective mean powers of 0.0003 ± 0.00003 mV^2^/Hz and 0.0002 ± 0.00003 mV^2^/Hz without significant differences. The mean power of the GRL group during the recordings was 0.0006 ± 0.0001 mV^2^/Hz, being higher (*p* < 0.001) relative to the control and ethanol groups, and lower (*p* < 0.0001) compared to that of the CTL-exposed fish. The mean power for the group exposed to CTL was much higher (0.002 ± 0.0003 mV^2^/Hz) (*p* < 0.0001) than that of the control and ethanol groups.

The average power during recovery of the GRL group was 0.0005 ± 0.00003 mV^2^/Hz, i.e., higher (*p* < 0.05) than those of the control and ethanol groups, and lower (*p* < 0.0001) than those of the CTL-exposed fish in recovery (Figure 4C). 

**Figure 5:**
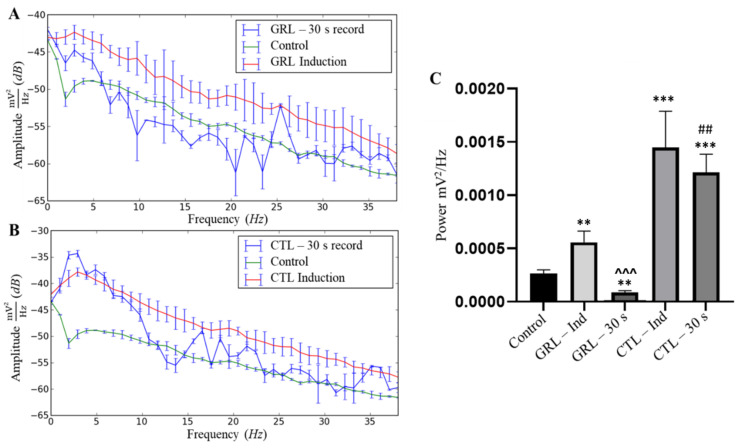
Power spectral density (PSD) of frequencies up to 40 Hz in tambaqui *C. macropomum* with comparisons among the last 30 s of the recordings (30 s), the control, total time of induction with geraniol (GRL—70 µL·L^−1^) (**A**), and total time of induction with citronellol (CTL—90 µL·L^−1^) (**B**). Differences in mean power among the control, ethanol, geraniol induction (GRL—Ind), citronellol induction (CTL—Ind), geraniol recovery (GRL—Rec), and citronellol recovery (CTL—Rec) groups are presented (**C**). * indicates statistical difference from the control group; ^ indicates statistical difference from GRL—Ind group; # indicates statistical difference from the CTL—Ind group (after ANOVA fol-lowed by Tukey’s test). Symbols in duplicate **, ## correspond to *p* < 0.001; Symbols in triplicate indicate ***, ^^^ correspond to *p* < 0.0001, (n = 9).

In the PSD of the GRL-exposed fish, a lower power was observed during induction within the last 30 s of the recording. The frequencies from 6 to 24 Hz were below those of the control, indicating a less intense signal captured by the electrode over the last 30 s of exposure (Figure 5A). For the CTL—30 s group, the last 30 s of the recordings demonstrated a greater power variation than those of the other groups, with oscillations below those of the control mainly between 13 and 14 Hz (Figure 5B).

The linear power distribution (Figure 5C) demonstrates mean values of 0.0003 ± 0.00003 mV^2^/Hz for the control group. In the GRL—Ind group, the mean power was 0.0006 ± 0.0001 mV^2^/Hz, i.e., higher than those of the control (*p* < 0.001) and GRL—30 s (*p* < 0.0001) groups, however, significantly lower relative to that of the CTL—induction group. For the GRL—30 s group, the tracings were more stable, showing a mean power of 0.00008 ± 0.00002 mV^2^/Hz, which was lower (*p* < 0.001) relative to that of the control. 

On the other hand, the CTL—Ind group had an average power of 0.001450 ± 0.0003388 mV^2^/Hz, differing from those of the control (*p* < 0.0001) and CTL—30 s (*p* < 0.001) groups (0.001216 ± 0.0001703 mV^2^/Hz) (Figure 5C). 

## 4. Discussion

Our results showed that the amplitudes of the EEG tracings observed in the control and ethanol groups were low, and the frequency spectrogram indicated that energy intensity was predominant at frequencies below 5 Hz throughout the recordings. On the other hand, fish exposed to GRL and CTL showed EEG tracing patterns of excitability, which were more persistent and rather pronounced in the latter. 

The monitoring of the brain activity through EEG is a tool for assessing the level of neuronal depression, as it will make it possible to verify the drug action at a central level [1,2,20,21]. Additionally, the analysis of the frequency spectrogram allows for the understanding of how the signal recorded by the EEG is composed, i.e., encompassing the amplitude, intensity, and frequency of the waves. Furthermore, the spectrogram can serve as an indirect assessment of the nociceptive and anaesthetic component generated in response to external stimuli that may be harmful to fish, since many phytoconstituents have demonstrated action on the CNS, including sedative, antinociceptive, and neuroprotective effects [21,22]. 

The analysed PSD, during the final 30 s of induction, showed that fish exposed to GRL presented reduced power relative to the control, from 8 to 24 Hz. These EEG changes seem to be well correlated with the anaesthetic effect of GRL, indicating that these frequencies are presumably a reflection of a general anaesthesia condition. Moreover, in terms of cardiac safety, although GRL caused a negative chronotropic effect in the heart of juvenile tambaqui, the sinus rhythm was preserved [12].

On the other hand, the alterations observed in the CTL-exposed fish, showed high mean amplitude of the tracings during the entire induction period, over the last 30 s, and during recovery. Although these responses were milder than those observed in fish exposed to eugenol [1], where animals showed tracing patterns similar to those of a convulsive episode, neuronal responses elicited by CTL seem not to support or comply with adequate general anaesthesia standards. Moreover, a recent study on the cardiac function of juvenile tambaqui exposed to CTL, described a marked bradycardia with arrhythmia and prolongation of the Q–T and R–R intervals and QRS complex duration, indicating a potential for atrioventricular blockade [12].

As a rule, it is widely accepted that the GABA_A_, NMDA receptors, and the voltage-gated channels (K^+^, Ca^2+^, and Na^+^) are the main targets of anaesthetics, being influenced in different ways [21,23,24]. The effects of monoterpenes can occur via different mechanisms due to their structural diversity. GRL has already been shown to have hypnotic-sedative action in tests with rats [21]. Presumably, the mechanism of action of GRL may be through the allosteric modulation of the GABA_A_ receptor, similar to that of menthol-induced anaesthesia, which is known to be a positive allosteric modulator of GABA_A_ [24]. According to this study, the chemical structure of menthol and an associated functional group (OH) trigger a positive modulation of the ion channels. Positive modulation of ligand-activated ion channels has a profound influence on neuronal activity, resulting in sedation and anaesthesia [24]. Such sedative effects of monoterpenes can be explained by their interaction with the GABA_A_ receptor [21]. The mechanism of action involved in GRL anaesthesia in fish is yet to be elucidated.

Regarding the mechanisms of action upon exposure to CTL, although it has already shown anticonvulsant and antinociceptive activity in rats, and is capable of inducing partial blockade of voltage-gated Na^+^ channels, which in turn are essential in regulating the excitability of neurons, and although such a blockade may facilitate the activation of the descending inhibitory pathway [22], our findings showed that this substance was not capable of promoting an adequate anaesthetic effect based on the EEG response attained.

Due to the complexity in capturing neuronal signals and the different monoterpene mechanisms of action, these compounds can elicit different EEG responses. Despite the recent advances in research relating the effects of various compounds on the fish brain activity [1,2,25,26,27], no studies are available to date, for instance, on the frequency bands and their correlation with general anaesthesia in fish. These are future research arenas that should be explored to best clarify how the fish brain responds to general anaesthesia. 

EEG wave patterns have not yet been systematically described for anaesthetised fish. Moreover, the route of the anaesthetic administration differs from that used for other upper vertebrates. However, it is already commonplace that such substances are capable of promoting important alterations in the CNS, which are reflected by distinct tracing patterns. Our results underscore the importance of the EEG in the evaluation of anaesthetic candidates prior to their broad recommendation for use in fish. 

In conclusion, both GRL at 70 µL·L^−1^ and CTL at 90 µL·L^−1^ promoted clear changes in the electroencephalographic tracings of juvenile tambaqui, *C. macropomum*. GRL led to characteristic neuronal effects of general anaesthesia, whereas CTL determined a more persistent brain excitability during induction, showing a more irregular wave oscillation pattern. While we endorse the use of geraniol as a novel and suitable general anaesthetic for fish, citronellol seems to have clear limitations for the purpose of fish anaesthesia as it did not lead to an adequate reduction in CNS activity.

## Data Availability

Data will be made available from authors upon reasonable request.
